# Computed tomography-guided core needle biopsy of lung lesions: Diagnostic yield and correlation between factors and complications

**DOI:** 10.3892/ol.2013.1680

**Published:** 2013-11-12

**Authors:** YING WANG, WENTAO LI, XINHONG HE, GUODONG LI, LICHAO XU

**Affiliations:** Department of Radiology, Fudan University Cancer Hospital, Shanghai 200032, P.R. China

**Keywords:** CT-guided, core needle biopsy, pneumothorax, hemorrhage, core needle biopsy diagnosis, final diagnosis

## Abstract

The aim of the present study was to determine the diagnostic accuracy of computed tomography (CT)-guided core needle biopsy (CNB) and to retrospectively analyze the correlation between the factors and complications of the procedure. Between January 2009 and June 2010, CNB was performed on 345 lung lesions in 343 patients. These patients were then followed up for at least two years. The sensitivity, specificity, accuracy, positive predictive value (PPV) and negative predictive value (NPV) of the CNB diagnoses were calculated. The correlation between factors, such as smoking, positoin and maximal diameter, and the complications of pneumothorax and hemorrhage was analyzed by χ^2^ test. The sensitivity, specificity, accuracy, PPV and NPV of the CNB diagnoses were 97.3, 100, 97.7, 100 and 87.7%, respectively. A statistically significant correlation was found between pneumothorax and the factors of smoking (P=0.015) and position (P<0.01) and length of the needle in the normal parenchyma (P=0.011), as well as between hemorrhage and the maximal diameter (P=0.005) and length of the needle in the normal parenchyma (P<0.01) and the frequency of needle adjustments (P<0.01). A CT-guided core needle biopsy of the lung lesions provides a high diagnostic yield. Smoking, the decubitus position and a longer length of the needle in the normal parenchyma were found to represent risk factors for a pneumothorax. In addition, a small diameter and longer length of the needle in the normal parenchyma and a more frequent adjustment of the needle were poor predictive factors of hemorrhage.

## Introduction

With the development of computed tomogrphy (CT), an increasing number of lung lesions are detected. It has been reported that >50% of resected pulmonary nodular lesions are related to malignancy; therefore, the requirement for rapid and definite diagnoses of lung lesions has been stressed (1)CT-guided needle biopsy has become the dominant method for obtaining tissue samples from lung lesions in order to obtain a pathological diagnosis (2). The procedure is generally regarded as safe, but pneumothorax, hemorrhage and other rare complications may occur. In addition, the reliability of a benign result remains controversial (3). The objective of the current study was to determine the diagnostic accuracy of CT-guided core needle biopsy (CNB) and to retrospectively analyze the correlation between factors and complications of the procedure.

## Materials and methods

### Study population

Between January 2009 and June 2010, a CNB was performed on a total of 343 patients with 345 lung lesions in 345 sessions. Among them, two patients received a second CNB, as the size of the tissue sample was not large enough to obtain pathological results. These patients were then followed up for at least two years. The enrolled population consisted of 228 males (66.5%) and 115 females (33.5%). The patient age ranged between 17 and 86 years old (mean age, 60±18.3 years old). The study was approved by the ethics committee of Fudan University (Shanghai, China).

### Procedure

Prior to the procedure, the risks and benefits were explained to each patient and informed consent was obtained. Three interventional physicians, each with >5 years experience of needle biopsies, performed the procedures. All cases were performed using the Philips 64-slice spiral CT (120 kV, 250 mA and a 3-mm thickness; Philips Healthcare, Andover, MA, USA) for imaging guidance. The biopsy tool that was used was an automated biopsy gun (SuperCore™, Angiotech Pharmaceuticals, Inc., Vancouver, BC, Canada) with an 18-gauge needle. The patient lay on the CT table and the puncture point and access routine were determined by CT scan. Following local anesthesia, the needle was inserted through the skin and advanced to the lesion. Intra-operative CT scans were performed to confirm the position of the lesion and needle. Once the needle was located in an appropriate position within or near the lesion, the operator triggered the cannula to close, trapping the specimen in the sample notch. Next, the tissue specimen was removed from the notch and sent to the Department of Pathology (Fudan University, Shanghai, China). Immediately after the procedure, a chest CT scan was performed to evaluate procedural complications, including pneumothorax and hemorrhage. In addition, a chest radiograph was obtained the following morning. The baseline characteristics of the lesions and procedures are summarized in Table I.

### Classification of diagnoses and complications

The CNB results were divided into 4 categories: Atypical adenomatous hyperplasia (AAH), malignant, benign and undetermined (3). AAH is a premalignant lesion of lung adenocarcinoma. Although AAH is not ranked as malignant, this category commonly undergoes carcinomatous change.

Final diagnoses were divided into 3 categories: Malignant, benign and undetermined. Malignant tumors were defined by: i) Cancer-associated mortality occurring during the follow-up period; ii) lesions representing complete response, partial response or progressive disease to chemotherapy according to the Response Evaluation Criteria in Solid Tumors (4); and iii) a histopathological examination showing malignant tumor tissue in the specimen received at surgical resection. Benign tumors were defined by: i) Lesions that disappeared or decreased in size with conservative treatment; ii) patient exhibited a positive microbiological conclusion (3,5); or iii) a histopathological examination showing no malignant tumor tissue in the specimen from surgical resection. Undetermined tumors were defined as: i) Lesions treated by radiofrequency ablation or stereotactic irradiation (6); ii) a lesion that was stable in size at follow-up; or iii) a patient that could not be contacted for follow-up (Table II).

AAH and malignant were considered to be positive results and benign was considered a negative result. Positive CNB results were considered true positives if the final diagnosis was malignant or AAH. By contrast, results were considered false positives if the final diagnosis was benign. Negative CNB results were considered true negatives if the final diagnosis was benign and false negatives if the final diagnosis was malignant or AAH (Table III).

**Figure 1:**
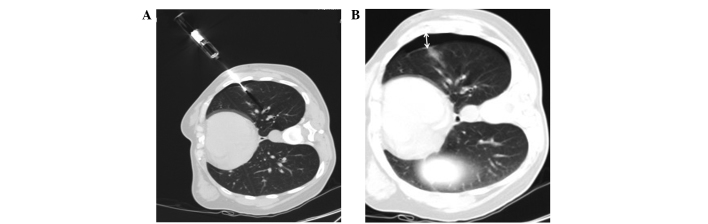
CT scan of a 48-year-old female. (A) Pure ground-glass opacity in the right lower lobe for CT examination. The core needle was in an appropriate position for the lesion. (B) Immediately after the biopsy, a mild pneumothorax with lung surface retraction (double arrow) of 1.6 cm was noted.

**Figure 2:**
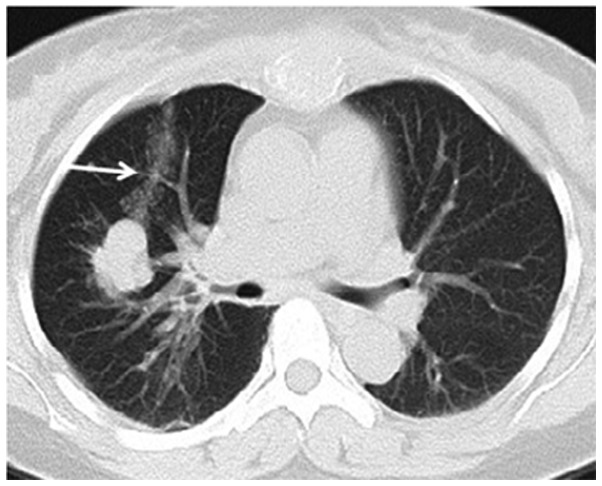
CT scan image. Mild hemorrhage with haziness along the needle tract.

**Figure 3:**
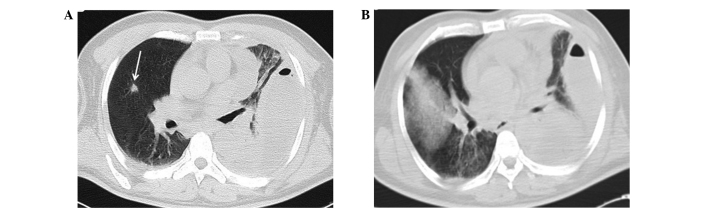
CT scan of a 54-year-old male. (A) Small nodule (arrow) in the right upper lobe with pleural effusion in the left thoracic cavity, demonstrated to be adenocarcinoma by biopsy and surgery. (B) Immediately after biopsy, a moderate hemorrhage with patchy haziness around the lesion was noted.

Pneumothorax was graded as mild (lung surface retraction of ≤2 cm; Fig. 1), moderate (lung surface retraction of 2–4 cm) and severe (lung surface retraction of ≥4 cm) (7). Hemorrhage was graded as mild (presenting as haziness along the needle tract; Fig. 2), moderate (hemoptysis of <30 ml or occurrence of patchy haziness around the lesion in CT; Fig. 3) and severe (hemoptysis of ≥30 ml or hemothorax).

### Statistical analysis

Data were analyzed with SPSS 19.0; the χ^2^ test was used for the statistical analysis. The analysis of pneumothorax and hemorrhage was based on the 345 CNB sessions not the 343 patients. P<0.05 was considered to indicate a statistically significant difference.

## Results

CNB diagnoses included 66 benign cases, 3 AAH cases, 256 malignant cases and 20 undetermined cases; final diagnoses consisted of 52 benign cases, 267 malignant cases and 26 undetermined cases. Final diagnoses were obtained based on the following characteristics: 11 (3.2%) patients succumbed to cancer-associated mortalities; 64 (18.7%) surgical specimens, 12 (3.5%) positive cultures; 193 (56.2%) positive responses to chemotherapy; 37 (10.8%) tumors disappeared or decreased in size with conservative treatment; 8 (2.3%) lesions were treated with radiofrequency ablation (n=2) or stereotactic irradiation (n=6); 5 (1.5%) lesions were stable during the follow-up period; and 13 (3.8%) patients could not be contacted for follow-up. Following exclusion of the undetermined CNB and final diagnoses (n=35) (8), the number of true positives, false positives, true negatives and false negatives were 253, 0, 50 and 7, respectively. The sensitivity, specificity, accuracy, positive predictive value (PPV) and negative predictive value (NPV) were 97.3, 100, 97.7, 100 and 87.7%, respectively.

In the present study, the overall complication rate was 50.4%. There was a total of 60 (17.5%) cases of pneumothorax in the 343 patients. Among those, 52 (15.2%) were mild, 6 (1.7%) were moderate and 2 (0.6%) were severe. Only 5 (1.5%) required placement of chest tubes. There were 113 (32.9%) cases of hemorrhage in this study. Of those, 78 (22.7%) were mild hemorrhage, 35 (10.2%) were moderate hemorrhage. No severe hemorrhage occurred. One of the patients with moderate hemorrhage complained of chest pain and chest tightness 8 h after the biopsy; the chest radiograph indicated a parenchymal hemorrhage and hemocoagulase was administrated. Another patient developed hypotension with syncope, and resuscitation and hemocoagulase administration improved the individual’s condition. All other hemorrhages were self-limited.

The correlation between factors and complications was determined, and a statistically significant correlation was found between pneumothorax and the factors of smoking and the position and length of the needle in the normal parenchyma (P=0.011). In addition, a statistically significant correlation existed between hemorrhage and the maximal diameter (P=0.005) and length of the needle in the normal parenchyma (P<0.01) and the frequency of needle adjustments (P<0.01). The correlations between factors and complications are shown in Tables IV and V.

## Discussion

In the present study, the incidence of pneumothorax was 17.5%, with 1.5% requiring chest tube drainage. These results are comparable to prior studies (9–11). The decubitus position was found to result in more pneumothoraxes than the supine and prone positions. We hypothesize that the decubitus position separates the parietal and visceral pleura more than the other positions and therefore, air is more likely to enter the pleural cavity as the needle is taken out. Moreover, the majority of lung lesions were found to be located in the middle or inner zone of the lung in the present study. In the biopsy of lesions located in the middle or inner zone of the lung, it is more difficult to keep away from the interlobar pleural; if it is damged, it is more likely to result in pneumothorax. O’Neill *et al*(12) reported that rapidly (within 10 sec) rolling the patient over to the biopsy-side-down position following needle-out reduced the rate of overall pneumothorax and pneumothorax necessitating a drainage catheter. Moreover, smoking, the decubitus position and a longer length needle in the normal parenchyma were risk factors for a pneumothorax. It is generally considered that a pneumothorax is the most common complication of a needle biopsy of the lung (11,13). However, in the present study, the incidence of hemorrhage (32.9%) was higher than pneumothorax (17.5%) and also higher than the 1–27% reported in the literature (7,11,14). This may be since a pneumothorax is easier to identify in chest radiographs and its obvious symptoms are apt to attract notice. By contrast, mild hemorrhage is difficult to identify in chest radiographs. Furthermore, the current data was based on the presence of haziness on CT images and hemoptysis, instead of hemoptysis only. The results showed that a small diameter and longer length of the needle in the normal parenchyma and more frequent adjustments of the needle were poor predictive factors of hemorrhage.

With regard to pathological yield, in the present study, the false positive and false negative rates and the PPV and NPV were 0, 2.7, 100 and 87.7%, respectively. When a malignant diagnosis is identified by needle biopsy, the clinical decision-making process is generally straightforward due to the extremely low false-positive rates (0.0–1%) (3,15). However, when a benign diagnosis is obtained, there is clinical uncertainty over how to proceed, as a number of these lesions may prove to be malignant (false negatives). Previous studies have evaluated the outcomes following a benign biopsy and have found false negative rates that vary widely (2–54%) (16–18). Combining the results of the present and relevant previous studies, we recommend that patients with benign CNB diagnoses undergo repeat imaging for ≥2 years to document the stability or resolution of the lesions. If the nodule grows, a repeat biopsy (19–21) or resection (22) may be required to obtain a definitive diagnosis.

The main limitations of the present study were as follows: Firstly, in the analysis of the correlation between factors and complications, single factors were considered individually without excluding the affect of other factors. Secondly, a small number of patients were lost for follow-up and a two-year follow-up may not have been sufficient.

In conclusion, CT-guided core needle biopsy of the lung lesions provides a high diagnostic yield. Smoking, the decubitus position and a longer length of needle in the normal parenchyma were risk factors for pneumothorax. In addition, small diameter, a longer length of needle in the normal parenchyma and more frequent adjustments of the needle were poor predictive factors of hemorrhage.

## Figures and Tables

**Table I:** Characteristics of lesions and procedures.

Characteristic	n (range)
Maximal diameter, mm	41.4 (8–134)
Lesion location	
Lobe	
Upper	178
Middle	28
Lower	139
Zone	
Outer	54
Middle	114
Inner	177
Patient position	
Supine	106
Prone	192
Decubitus	45
Frequency of needle adjustments	1.6 (1–7)
Distance from skin puncture to needle tip, mm	73.0 (4.8–139.6)
Distance from pleural puncture to needle tip, mm	34.4 (8.9–87.8)
Length of needle through normal parenchyma, mm	14.8 (0.0–59.7)

**Table II:** CNB and final diagnosis.

	Final diagnosis		
	
CNB diagnosis	Benign	Malignant	Undetermined
Benign	50	7	9
AAH	0	3	0
Malignant	0	250	6
Undetermined	2	7	11

CNB, core needle biopsy; AAH, atypical adenomatous hyperplasia.

**Table III:** Positive and negative diagnosis.

	Final diagnosis		
	
CNB diagnoses	Positive	Negative	Total
Positive	253	0	253
Negative	7	50	57
Total	260	50	310

Undetermined CNB diagnoses and final diagnoses were excluded from calculation. CNB, core needle biopsy.

**Table IV:** Correlation between factors and pneumothorax.

Characteristic	Pneumothorax, n	Non-pneumothorax, n	P-value
Gender			0.956
Male	40	189	
Female	21	95	
Age, years			0.986
17–49	12	58	
50–69	34	146	
70–86	15	80	
Smoking status			0.015^a^
Non-smoker	17	44	
Current or ex-smoker	20	126	
Unknown	24	114	
Patient position			0.000^a^
Supine	16	89	
Prone	25	170	
Decubitus	20	25	
Lesion location			
Lobe			0.568
Upper	29	158	
Middle	5	25	
Lower	27	101	
Zone			0.115
Outer	14	41	
Middle	24	86	
Inner	23	157	
Effusion			0.181
No	56	246	
Yes	5	38	
Maximal diameter, cm			0.605
≤1	2	4	
1–3	22	89	
3–5	26	107	
>5	11	84	
Length of needle through normal parenchyma, mm			0.011^a^
<1	14	124	
1–3	31	102	
≥3	16	58	
Needle-hilus angle, °			0.156
0–30	22	95	
30–60	32	125	
60–90	7	63	
Needle adjustments			0.285
1	38	176	
2–3	14	83	
≥4	9	25	
Operator/s			0.210
1	34	136	
2	15	60	
3	12	88	

a Statistically significant difference.

**Table V:** Correlation between factors and hemorrhage.

Characteristic	Hemorrhage, n	Non-hemorrhage, n	P-value
Gender			0.747
Male	75	154	
Female	40	76	
Age, years			0.455
17–49	27	43	
50–69	55	125	
70–86	33	62	
Smoking status			0.697
Non-smoker	20	41	
Current or ex-smoker	52	94	
Unknown	43	95	
Patient position			0.211
Supine	32	73	
Prone	72	123	
Decubitus	11	34	
Lesion location			
Lobe			0.466
Upper	65	122	
Middle	7	23	
Lower	43	85	
Zone			0.336
Outer	14	41	
Middle	36	74	
Inner	65	115	
Effusion			0.134
No	10	33	
Yes	105	197	
Maximal diameter, cm			0.005^a^
≤1	5	1	
1–3	43	68	
3–5	45	88	
>5	22	73	
Length of needle through normal parenchyma, mm			0.000^a^
<1	18	120	
1–3	49	84	
≥3	48	26	
Needle-hilus angle, °			0.465
0–30	36	81	
30–60	55	102	
60–90	23	47	
Needle adjustments			0.000^a^
1	53	161	
2–3	48	59	
≥4	14	20	
Operator/s			0.797
1	55	115	
2	24	51	
3	36	64	

a Statistically significant difference.
